# Dual-Polarization Observations of Slowly Varying Solar Emissions from a Mobile X-Band Radar

**DOI:** 10.3390/s17051185

**Published:** 2017-05-22

**Authors:** Marco Gabella, Andreas Leuenberger

**Affiliations:** MeteoSwiss, via ai Monti 146, Locarno-Monti CH-6605, Switzerland; andreas.leuenberger@meteoswiss.ch

**Keywords:** meteorological radar receiver, absolute calibration, dual-polarization, solar radio noise, antenna gain (with radome), slowly varying component of the solar emission at 3.2 cm

## Abstract

The radio noise that comes from the Sun has been reported in literature as a reference signal to check the quality of dual-polarization weather radar receivers for the S-band and C-band. In most cases, the focus was on relative calibration: horizontal and vertical polarizations were evaluated versus the reference signal mainly in terms of standard deviation of the difference. This means that the investigated radar receivers were able to reproduce the slowly varying component of the microwave signal emitted by the Sun. A novel method, aimed at the absolute calibration of dual-polarization receivers, has recently been presented and applied for the C-band. This method requires the antenna beam axis to be pointed towards the center of the Sun for less than a minute. Standard deviations of the difference as low as 0.1 dB have been found for the Swiss radars. As far as the absolute calibration is concerned, the average differences were of the order of −0.6 dB (after noise subtraction). The method has been implemented on a mobile, X-band radar, and this paper presents the successful results that were obtained during the 2016 field campaign in Payerne (Switzerland). Despite a relatively poor Sun-to-Noise ratio, the “small” (~0.4 dB) amplitude of the slowly varying emission was captured and reproduced; the standard deviation of the difference between the radar and the reference was ~0.2 dB. The absolute calibration of the vertical and horizontal receivers was satisfactory. After the noise subtraction and atmospheric correction a, the mean difference was close to 0 dB.

## 1. Introduction

The use of extraterrestrial radio noise sources as calibration aids has been reported in the literature since the 1950s, with the aim, in particular, of evaluating the performance of radio telescopes [1,2,3]. As far as the calibration of weather radar receivers is concerned, Whiton et al. [4] first introduced the idea of using the Sun in 1976, and after further discussions on the topic, Frush presented a practical methodology in Zurich in 1984 [5]. In 1989, Pratte and Ferraro [6] presented the first quantitative comparison of S-band horizontal polarization radar-derived solar flux values at the National Center for Atmospheric Research (NCAR) and accurate reference measurements acquired by several observatories (e.g., the Dominion Radio Astrophysical Observatory (DRAO) of the Canadian National Research Council and National Oceanic and Atmospheric Administration solar- terrestrial data acquired by the US Air Force Radio Solar Telescope Network (RTSN) in Palehua, Sagamore Hill, Learmouth. According to the observations of the NCAR CP-2 radar, where DRAO measurements were used as a reference, the standard deviation of the multiplicative error that affected 14 retrieved values was 0.14 dB. The 14 semi-automated radar observations that were considered had been acquired between November 1987 and August 1988, which was an active solar period. Therefore, using their semi-automated sun-tracking technique, a radar operator was able to perform the data acquisition phase in approximately 15 min. Later, Pratte, Ferraro and Keeler further extended the methodology, but, probably because of the long off-line time (three to five sun-tracking scan programs were generally run and the median value was kept as the observation of the specific day), it only became popular a few decades later, when the data acquisition time became considerably shorter. The method was eventually transferred to the United States weather radar network by Ice and colleagues [7] for use with the S-band, polarimetric upgraded WSR-88D radar. A detailed quantitative comparison of C-band dual-polarization radar-derived solar flux values and accurate reference measurements provided by DRAO was also published in 2015 [8]: the standard deviation of the difference between 7 radar observations, measured during the Site Acceptance Tests of the MeteoSwiss Weissfluhgipfel radar at an altitude of 2850 m, and the (frequency transformed) DRAO reference was as small as 0.09 (0.13) dB for the vertical (horizontal) polarization. These preliminary results were so encouraging and promising that MeteoSwiss decided to implement a fast, semi-automated sun-tracking technique, which has also been applied to the other four polarimetric operational weather C-band radars [9]. With such a semi-automated sun-tracking technique, data acquisition is performed in just a few minutes. Remarkably, this methodology tackles both the relative (standard deviation of the error around the mean) and absolute calibration of the receiver for the first time [8,9]. The major limitation of the sun-tracking technique is that it requires the weather radar to be off-line for a few minutes.

In order to overcome this limitation, a complementary on-line technique that automatically detects and analyzes signals from the sun which are stored in the polar volume radar reflectivity data acquired during the operational weather scan program was conceived [10,11,12]. This operational monitoring method has been implemented successfully to determine the electromagnetic antenna pointing [10], and assess receiver stability [11] and the differential reflectivity offset [12]. The results of such a method were first applied successfully during a period of quiet solar flux activity in 2008 [11,12] and then to an active solar period in 2014 [13]. All the Swiss radars were able to capture and describe the ~27-day period of the slowly varying solar component. The technique has allowed a relative calibration and a mutual inter-comparison to be made between the vertical and horizontal channels and has the great advantage of requiring no interruption of the weather surveillance. A recent work by Huuskonen et al. [14] presents further developments of the method: for the daily monitoring of the differential reflectivity bias of polarimetric weather radars, an improved quality control is applied to the solar signals detected during the operational scanning, which efficiently removes rain and clutter contaminated gates occurring in the solar hits.

In short, the focus of most of the sun related papers [4,5,6,7,8,9,10,11,12,13,14] presented in this introduction has been on relative calibration: horizontal and vertical polarization signals have been (mutually) compared and evaluated versus the DRAO reference signal, mainly in terms of standard deviation of the difference between radar-retrieved and reference flux values. Nevertheless, some authors [8,9] have presented a novel method aimed at the absolute calibration of the dual-polarization weather radar receivers. The two major aims of this paper are: (A) to present the application of the aforementioned methodology [8,9] to another frequency band, namely the X-band (~10 GHz); and (B) to show that it is possible to follow the slowly varying component of the solar radiation with a directive antenna and a sensitive radar receiver even at ~3 cm. The spectral range over which the slowly varying solar component is “significant”, compared to the quiet radio flux, is between approximately 57 and 2 cm, but the effect is maximum at around the geometrical average of these two boundaries (S-Band) and minimum at the edges of such an interval.

This paper is organized as follows: Section 2 briefly describes the accurate extraterrestrial source of radio noise that has been used for the absolute calibration of the dual-polarization radar receivers i.e., the sun. Section 2.1 briefly describes the reference values that were measured accurately at S-band three times a day by the DRAO of the Canadian National Research Council. Section 2.2 presents the formula, assumptions and limitations of the technique used to transform the S-band values of the solar flux to the frequency band (X-band) of our mobile weather radar. Section 3 presents the core of the paper: the extension of our method to the absolute calibration of the weather radar receivers from the C-band [8,9] to the X-band. Section 4 presents the results, in graphical form, and in Section 5 the results are discussed in further detail. The quantitative results (as well as the reference values made publicly available by the DRAO of the Canadian National Research Council) are listed in tabular form in Appendix A.

## 2. Radio Emission Properties of the Sun

The Sun’s radio output varies considerably in terms of time and frequency. Solar conditions are often categorized as quiet, disturbed and active. More information can be found in [15] as well as in Section 1 of the extended abstract [16] presented at the WMO TECO conference in Madrid.

### 2.1. Accurate Measurements of the Solar Flux at the S-Band: The DRAO Reference

The solar flux is continuously monitored at the Dominion Radio Astrophysical Observatory (DRAO) at a 10.7 cm wavelength, that is inside the so-called S-band. The observatory has been located near Penticton in British Columbia, Canada, since 1990 and characterized by low interference levels at the decimeter and centimeter wavelengths; the quality of the environment is maintained by means of local, provincial and federal protection [17]. Details regarding the measurements and the hardware (antenna, receiver, reference noise source, …) can be found in Section 4 in [17]. DRAO measurements are more accurate than the values retrieved using weather radar observations. It is worth noting that: (1) they last one hour (instead of a minute); (2) the antenna half power beam width is much larger than the solar disk; (3) DRAO measurement scheme waits at an estimated position for the sun to pass by, while Sun-tracking, which is scheduled every 30 min (see Section 3.1) may be affected by variable ground noise effect.

### 2.2. Transforming the 10.7 cm DRAO Solar Flux Measurements to the Corresponding X-Band Values

In order to be correct and rigorous, we should collect dedicated absolute measurements of the solar flux at the 3.2 cm wavelength rather than transforming the reference S-band values into the X-band. However, a transformation equation that makes use of the remarkable stability of the blackbody spectrum of the slowly varying component of the solar activity can be used if one is ready to accept some degree of uncertainty in the calibration procedure. The equation is a simple linear transformation:

*F*_λ_ = *p*_λ_(*F*_10.7_ − q_10.7_) + *P*_λ_, (1)

where *p*_λ_ represents a scaling factor and q_λ_ represents the quiet sun flux density of the desired wavelength and the q_10.7_ value is the quiet solar flux density at the 10.7 cm reference wavelength. The rationale behind this equation is described in detail in Section 5 of [17]. Using the values listed in the table shown in Section 5 of [17], it is possible to derive the solar flux at the λ = 3.2 cm wavelength, *F*_3.2_.

From a radiometric viewpoint, the flux densities observed in radio astronomy are spectral irradiances, dimensions for which are power per unit bandwidth and per unit equivalent area. These values are typically very small if expressed using the SI units, hence they are expressed in the solar flux units (sfu), where 1 sfu = 10^−22^ W∙m^−2^∙Hz^−1^. According to [17], the quiet solar flux density at the reference frequency is q_10.7_ = 64 sfu, as shown inin Equation (2).

*F*_3.2_ = 0.69·(*F*_10.7_ − 64) + 255. (2)

## 3. Deriving Solar Flux Values Using Observations of a Mobile Radar Working at the X-Band

### 3.1. The Swiss Confederation Dual-Polarization X-Band Weather Radar

A few years ago, the Swiss Confederation acquired an innovative, state-of-the-art, mobile, dual-polarization, Doppler weather radar to assess the effect of precipitation on the performance of precision approaching radars (PAR). MeteoSwiss is currently operating the X-band radar in parallel with the PAR site acceptance tests at designated locations. The products generated by the weather radar are used to assess the state of the atmosphere within and around the PAR cells. Refer to Section 2.3 of [16] for further details.

The system is provided with an accurate antenna control unit that allows tailored, powerful and precise 3D scanning operations to be conducted. Hence, it was possible to implement not only conventional, meteorological scan programs, but also innovative ones. The following implemented scan programs are worth mentioning: (A) 10-min co-located PAR cell monitoring; (B) 2-min adaptive track of thunderstorm cells; (C) 30-min adaptive and fully automatic Sun-tracking. As far as (A) and (B) are concerned, more information can be found in [18,19], respectively. As for (C), it is worth noting that, because of our adaptive scan, the sun is automatically tracked every half hour, just after and before the ordinary meteorological scans, which is, Polar Plane Indicator (PPI) and Range Height Indicator (RHI). The system is also equipped with a powerful Radar Signal Control and Processing software (Ravis^®^) that allows, among many other features, an accurate manual and/or automatic Sun-tracking. However, even in automatic mode, the Ravis software needs an operator to launch the sun raster scan, while our adaptive scan is totally unmanned. Note that during the Sun-tracking, Ravis turns the transmitter off, while during our 30-min adaptive scan (interleaved between PPI and RHI) the transmitter is on. The list of benefits associated with this mobile system is long and the spectrum of possible atmospheric observations is wide, e.g., [18,19,20,21].

Because of the low receiver losses, the presence of a Low Noise Amplifier (LNA) at the first stage of the receiver chain and a directive antenna (HPBW = 1.3°), the system is sensitive enough to detect the solar flux, although with a relatively poor Signal-to-Noise ratio. The ratio between a measurement with the antenna beam axis pointed towards the center of the sun (Signal + Noise) and a noise measurement acquired at high elevation is in fact around 4 dB. This implies that noise subtraction reduces the raw observations by approximately 1.8 dB (as can be seen in Appendix A, Table 1, which lists the results of the retrieval for both the original observations and after noise subtractions).

From a practical viewpoint, in order to precisely quantify the amount of solar power that is reaching the LNA at the entrance of the radar receiver (Rx) during a clear sky day, it is necessary to know the equivalent area of the radar antenna and the Rx losses (including dry radome losses) along the path. These characteristics are listed in Table 1 for both polarization channels. It should be noted that the antenna performance is expressed in terms of maximum gain, as measured by the manufacturer at the test-range. In order to derive the equivalent area, A_eq_, in m^2^ from the (horizontal and vertical polarization) gain measured at the test range, Equation (5) is used.

### 3.2. The X-Band Radar Calibration Concept and the Conversion of the Solar Signal from Log-Transformed Analogue-Digital-Units (dBadu) to Solar Flux Units (dBsfu)

During the 30-min automatic, dedicated Sun-tracking, the maximum possible amount of incoming spectral irradiance from the sun hits the parabolic antenna reflector (the antenna beam axis is pointed towards the center of the sun). It is important to note that this incoming signal is the sum of the solar power plus an unknown noise power so that the corresponding value at the output of the A/D converter S_adu_, which is expressed in analog-to-digital units (adu), contains both the informative and the parasitic signal. The amount of spectral power that reaches the reference point, which is the Rx front-end at the LNA input, depends on the antenna gain and receiver chain (waveguides, circulator, T-R limiter, …) plus radomelosses. The given amount of received power (mW), which also depends on the bandwidth of the Rx transfer function, is transformed to a given digital unit value by the linear A/D converter. In short, the sun(+Noise) signal detected by the radar induces a given level at the output of the A/D converter, where S_dBadu_ is the log-transformed value in the analog-to-digital-units (dBadu) at the output of the A/D converter. The radar establishes the exact power of any signal by comparing it with a known reference power: this is the key radar calibration concept (see Section 3.2.1). Once the incoming solar power at the reference point has been assessed by means of electric calibration it is possible to retrieve the corresponding log-transformed incident solar spectral irradiance at the 3.2 cm wavelength, I_3.2_, that hits the antenna. This can be achieved through two additional steps together with a few conceptual assumptions (Section 3.2.2 and Section 3.2.3). As stated, this original, “raw” spectral irradiance has two components: the solar radio noise (which is informative for us) plus an unknown noise component. Every 30 min, in addition to the (Sun + Noise_unk_) component (antenna beam axis hitting the center of the sun), a simultaneous estimate of the noise is retrieved by measuring an angular offset of 2° with respect to the center of the solar disc. This estimated signal is then subtracted from the original, “raw” signal in order to get a better estimate of the solar power. The closer is the estimated noise to the unknown noise, the better is the estimate of the true solar signal after noise subtraction. For both the noise and the sun+noise, the estimate is based on 11,000 samples, namely 1100 (100) samples in range (Azimuth): the Azimuthal resolution is 0.1° (PRF = 2000 Hz, angular velocity set to 2°/s), while the range resolution is 50 m (echoes from 20 to 75 km are used).

#### 3.2.1. Converting the Solar dBADU Level into (Log-Transformed) Power (dBm) at the Entrance Reference Point

Owing to the linear behavior of the radar receiver over a huge dynamic range, the incoming solar value, S_dBadu_, in dBadu, can be converted into (log-transformed) power (in dBm) after a simple comparison with the corresponding value of the injected reference signal. In the case of the mobile X-band radar operated by MeteoSwiss, the reference signal is obtained from a monochromatic signal provided by an Internal Test Signal Generator (ITSG): ITSG_rp_ is the injected, reference power value in dBm, while ITSG_dBadu_ is the corresponding level at the output of the A/D converter. Let S_rp_ be the power of any received solar signal that one would like to determine, and S_dBadu_ its corresponding level, as read at the output of the A/D converter. Then one obtains:

S_rp_ = ITSG_rp_ + S_dBadu_ − ITSG_dBadu_, (3)

where [ITSG_rp_] = [S_rp_] = dBm, while [ITSG_dBadu_] = [P_dBadu_] = dBadu. For the Payerne field experiment, ITSG_rp_ was −56.2 (−56.7) dBm, for the horizontal (vertical) channel, while the corresponding levels at the output of the A/D, ITSG_dBadu_ were 66.4 and 64.9 dBadu, respectively.

**Figure 1:**
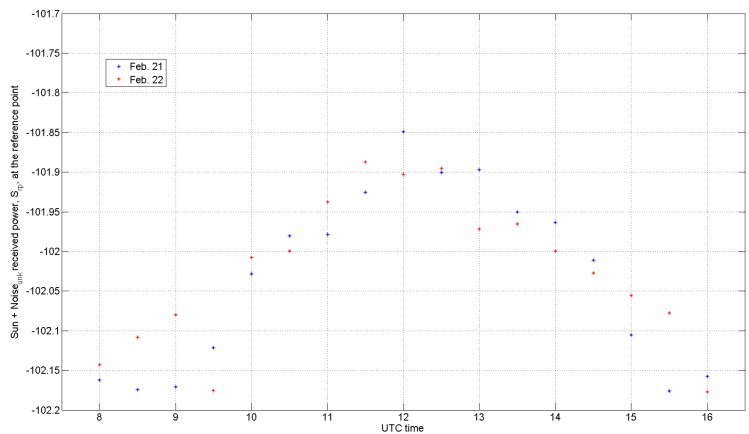
Signal received from the Sun (horizontal channel), S_rp_, at the reference point, which is at the entrance of the Low Noise Amplifier (LNA) (see the left-hand term in Equation (3)) over two consecutive winter days. Since the attenuation of the Earth’s atmosphere has not been corrected, a maximum can be observed in correspondence to the largest angle of elevation value.

Figure 1 shows examples of S_rp_ acquired using the horizontal channel every 30 min during two consecutive winter days. The observations show similar and representative time trends: since attenuation by the Earth’s atmosphere is not corrected, a maximum is expected around mid-day in correspondence to the largest angle of elevation value. However, it is worth recalling that the signal variability also depends on the variation of the temperature inside the antenna-mounted receiver box. Our procedure to read the daily solar maximum is very simple: we just keep the daily 2nd MAX between 10 and 14 UTC and round it to the nearest 0.05 dB. Hence, our daily estimate of S_rp_ for both 21 and 22 February is −101.9 dBm.

1. As far as the source of the reference signal is concerned, it is important to note the following three limitations of the ITSG solution compared to the more advanced solution implemented in the dual-polarization MeteoSwiss weather radar network that has recently been installed in the framework of the Rad4Alp project [22]. The ITSG signal cannot be used for continuous monitoring of the receiver. It can only be injected on demand, as long as the radar is offline.
2. It is plausible that the sensitivity of the ITSG to temperature is not less than the Rx sensitivity itself (see, for instance, the considerations on atmospheric attenuation at the end of Section 3.2.3 and the variability observed in Figure 1).
3. The monochromatic signal of ITSG does not homogeneously fill the whole matched-filter band width.

The last two limitations are addressed in the Swiss Confederation weather radar network (Rad4Alp) by using a white signal, generated by a noise source (NS): as described in [23], the selected NS shows high stability and reproducibility, and its sensitivity to temperature is considerably smaller than the overall Rx amplifying chain. Furthermore, the NS white signal completely fills the whole matched-filter bandwidth. As for the first limitation, the present solution in the X-band radar does not allow a continuous monitoring of the Rx chain: the ITSG_rp_ and ITSG_dBadu_ terms in Equation (3) are both assumed to be constant. Instead, with the (C-band) Rad4Alp calibration concept, slightly different values in dBadu at the output of the A/D converter could correspond to the same injected reference value.

#### 3.2.2. Assessing the Unpolarized Solar Power in dBm at the Entrance of the Antenna Feed

The assessment of the measured unpolarized Solar signal at another reference point (outside the radome, and not yet captured by the radar antenna) requires the knowledge of three terms:

- the multiplicative factor from unpolarized solar radiation to dual-pol channels (dual-polarization loss);
- accurate knowledge of the Rx chain losses, L_Rx_, including dry radome attenuation for both polarizations;
- (finally,) since the solar disc is not seen with a constant antenna Gain due to the directive weather radar antenna, the Sun cannot be considered as a point source.

As far as the first term is concerned, the factor can easily and reasonably be assumed equal to 2 (that is, by adding 3 dB to the log-transformed scale). As for the Rx chain losses (including the dry radome losses), reference can be made to the measurements performed during the Site Acceptance Test (Locarno airport, autumn 2012); the values are listed in the first line in Table 1. Regarding the last point, since the half power beam width of the antenna is not much larger than the solar disk, the contribution of the outer areas of the disc are underestimated compared to the inner part; hence the observed flux density starts to deviate (decrease) from the integrated flux density observed when a broader-beam antenna (for instance, the DRAO antenna, whose HPBW is 4.5°) is used.

In principle, if the beam axis is pointed precisely towards the center of the disc and the solar disc is uniformly bright, it is possible to quantify such non-point-source, L_nps_. For instance, according to Section 7.1 in Reference [17] or Equation (13), page 161, in Section 2.d in Reference [11], if an apparent diameter of the radio sun equal to 0.57° and HPBW = 1.3° are assumed, L_nps_ = 10 Log (0.8954) = 0.29, which we have approximated to 0.3 dB. However, it is worth noting that the uniformly bright sSun disc condition is encountered often around the solar activity minimum, but more rarely elsewhere during the solar cycle.

The incident solar power at the antenna feed, P_ant_, can be derived simply (in an equation) as:

P_ant_ = S_rp_ + 3 dB + L_Rx_ + L_nps_ = S_rp_ + 3.3 dB + L_Rx_. (4)

where S_rp_ has been estimated using Equation (3). P_ant_ has the same dimension as S_rp_, namely [P_ant_] = dBm.

#### 3.2.3. Estimate of the Unpolarized Incoming Solar Spectral Irradiance, I_3.2_, in Solar Flux Units (where O_2_ Attenuation is Neglected)

As stated in Section 2.2, the DRAO reference values consist of the spectral power per unit of area, which is a quantity that is called irradiance in the radiative transfer theory. However, at microwave frequencies, it is expressed in the Solar Flux Units (SFU), where 1 SFU is equal to 10^−19^ mW∙m^−2^∙Hz^−1^. Hence, the Rx bandwidth (in Hz) and the antenna equivalent area, A_eq_ (in m^2^) should be known as precisely as possible for the final comparison in order to transform the estimated incident power at the antenna feed, given in dBm, P_ant_, into the incident spectral irradiance at 3.2 cm, I_3.2_, which in the same way as the DRAO reference, is expressed in dBsfu.

The equivalent area of the antenna is estimated from the (horizontal and vertical polarization) gain, *G*, measured at the test range, using the following equation:

(5) $$A_{eq}=\;G\frac{\lambda^{2}}{4\pi }$$

The uncertainties related to the assessment of the equivalent Rx bandwidth are (certainly) smaller than those associated with the estimation of the equivalent area: the bandwidth of the matched filter is 3.6 MHz for the typical transmitted pulse of the X-band radar (pulse width τ = 333 ns). However, recent investigations [24] have shown that the bandwidth should be extended to a value of 3.78 MHz in order to calculate the power of noise signals. This means a value of 65.77 dBHz on a log-transformed scale. In short, the third step necessary to transform the Rx dBadu into dBsfu is:

(6) $$I_{3.2}=P_{ant}+190\;dB-B_{dBHz}+10\;Log\left(\frac{4\pi }{\lambda^{2}}\right)-G_{dB}.$$

As a practical example, it can be seen, in Figure 1, that S_rp_ (2nd MAX and 0.05 dB rounding) was −101.90 dBm on both 21 and 22 February 2016 for H polarization. By applying Equations (4) and (6), it is possible to obtain an estimated solar flux (Sun + Noise) at the X-band equal to 26.06 dBsfu, which can be compared with the DRAO reference value obtained for those two days.

The next section deals with how a reasonable comparison can be performed. It is worth noting that although the DRAO references are corrected for clear sky tropospheric attenuation, our radar derived estimates are not compensated. The total atmospheric attenuation at the zenith for the X-band and for a radar site altitude of 500 m above sea level (such as in Payerne, Switzerland) is of the order of 0.1 dB (if a one-way attenuation of 0.013 dB/km at the sea level and an equivalent atmospheric height of 8.5 km are assumed). For the practical example shown in Figure 1, this would correspond to approximately 0.554 dB in the morning and afternoon (angle of Elevation of ~10°) and to 0.1992 dB at an angle of elevation of 30°: hence, a decrease in attenuation of ~0.35 dB could be expected, while an approximate difference of 0.2 dB can be observed in Figure 1. However, the clear-sky atmospheric attenuation at mid-day, which is smaller than 0.199 dB (angle of Elevation of the 2nd MAX is always larger/greater than 30°) is neglected in Section 4 and Section 5.

## 4. Results

This section presents the results obtained in 2016 when the X-band radar was deployed at the Payerne airport in Switzerland. The data set consists of 57 daily radar estimates, retrieved from 2 February to 30 September during days without precipitation. Such estimates are compared with the X-band transformed (see Section 2.2) DRAO reference values. As stated in Section 2.1, DRAO acquires accurate measurements of the Sun three times a day. Our “smoothed” reference has been derived, for each day presented in this section, by averaging the five closest-in-time DRAO values. It is worth noting that the variability of these five values is so small that the use of a decibel scale with two digits after the decimal point renders the “log of the mean” indistinguishable from the “mean of the log” for all of the analyzed days. Such a two-digit average of DRAO flux values is expressed in dBsfu: the values are listed in the 4th column in Table A1 (Appendix A), and are followed by a three-digit standard deviation, calculated using the 5 values on the logarithmic decibel scale. The first columns in Table A1 indicate the Julian day number in 2016, starting from day 33 (2 February) and ending on day 274 (30 September). The other four columns show the values retrieved using the horizontal and vertical channels for the nominal acquired values and the (estimated-)noise subtraction.

### 4.1. An Intuitive Visual Comparison to Assess the Performances at a Glance

**Figure 2:**
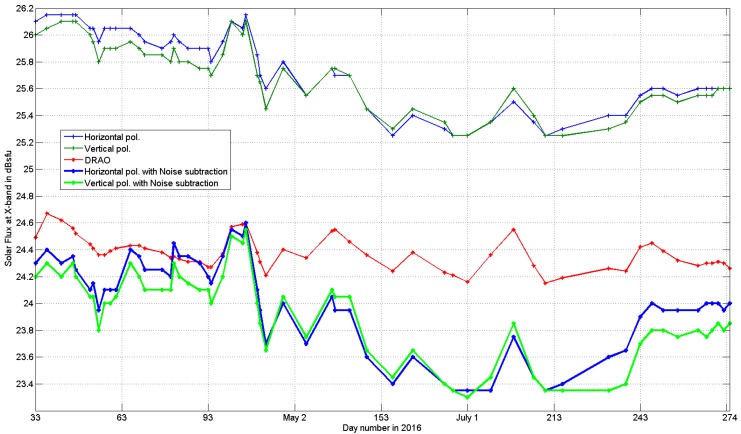
The red curve represents the daily solar flux values measured by the Dominion Radio Astrophysical Observatory (DRAO) in Canada at 10.7 cm and extrapolated to the X-band. The other four curves show the values retrieved by the Swiss Confederation mobile weather radar.

The fluctuations with time of the slowly varying component of the X-band solar spectral irradiance values listed in Table A1 are shown in Figure 2: the 1st column values are on the abscissa, while the other five columns are on the ordinate. The DRAO reference is in red and the radar-derived horizontal (vertical) values are in blue (green). It is clear that all the retrieved values (both from the original data and after noise subtraction) are able to capture and describe the (~27 day) periodic evolution of the microwave signal emitted by the sun, despite a relatively poor S/N ratio and the fact that the amplitude of the slowly varying component at 3.2 cm is only ~69% (see Equation (2)) of the maximum oscillation, which is observed at the S-band. To the best of our knowledge, this is the first time that the solar slowly varying component has been monitored at the X-band by dual-pol receivers. As it will be seen in Section 5, the retrieved values at 3.2 cm are far from the accurate monitoring of solar activity performed by DRAO: the explained variance between 57 data pairs is of the order of 55% (41%) for the vertical (horizontal) polarization (performances after having subtracted the estimated noise). The situation is obviously better at longer wavelengths: for instance, the explained variance by the C-band MeteoSwiss Albis radar during 220 days in 2014 was 83% (see Table 2 in [13]).

The DRAO reference and the radar retrieved curves both show four pronounced relative maxima in February, April, May and July. There is also agreement for the lowest values of the relative minima. As expected, given the relatively poor sun-to-noise ratio, the “raw” Sun + Noise values (upper thin blue and green curves) are not very close to the DRAO reference: on average, +1.38 (+1.32) dB can be observed for the horizontal (vertical) channel. After the noise subtraction, the average residual bias is reduced significantly: on average, −0.38 (−0.46) dB can be observed for the horizontal (vertical) channel, as seen in the 2nd line in Table 2.

### 4.2 Quantitative Comparison between the DRAO Reference and the Retrieved Radar Values

Sun + noise values cannot be used to assess the absolute calibration of the horizontal and vertical channels, because the noise contribution to the received signal is not negligible; for such a purpose, it is necessary to use the solar flux estimates after noise subtraction (the two lowest blue and green curves in Figure 2). The last two columns in Table 2 show the main statistical parameters of the retrieved estimates after noise subtraction, and these are comparable with the reference ones (DRAO, red curve). On average, both channels are well calibrated: the average underestimation is approximately 0.2 dB (it should be recalled that the atmospheric attenuation, which has not been accounted for here, is of the order of 0.2 dB), with the vertical channel being ~0.1 dB lower than the horizontal one (while the median value is practically the same).

As far as the dispersion around the average value of the curves shown in Figure 2 is concerned, the DRAO dispersion is the smallest, while the dispersion after noise subtraction is the largest; the dispersion of the sun+noise curves is somewhere in the middle, with the horizontal polarization being larger than the vertical one, in the same way as after noise subtraction (see the last line in Table 2).

## 5. Discussion

It was previously observed that “raw”, original data cannot be used for an accurate assessment of absolute calibration, because the noise contribution to the detected signal is not negligible. Noise subtraction is necessary as without doing that one would erroneously conclude that the X-band radar is positively biased by approximately 1.6 dB (plus atmospheric attenuation), while the radar estimates on average, are almost bias-free. However, this fact is only valid for the average value. The small residual negative bias is not constant as it can be over or underestimated. The dispersion of the difference can be assessed, for instance, by using the standard deviation. The results of such a procedure are given in Table 3, where shows: (1) the vertical polarization is slightly better than the horizontal one for both the the Sun + Noise values and after noise subtraction; (2) the standard deviation of the difference increases for both polarizations after daily noise subtraction. Hence, it seems better to subtract a constant noise value than trying to estimate and subtract it daily and subtract on a daily basis.

In order to confirm this important conclusion, an attempt could be made to quantify the agreement between the DRAO and the radar-retrieved (original vs. Noise-subtracted) estimates from another viewpoint, that is, in terms of correlation. Table 4 shows the explained variance, which is 100 times the square of the correlation coefficient between the DRAO reference and the radar estimates: again, the original (sun + noise) values give a better score for both polarizations. It is possible to conclude that it is better to subtract a constant noise value than a daily-retrieved one, even in terms of correlation. In other words, our estimate of the noise power that affects the solar signal is still not optimal: if it were possible to improve the estimate of the noise, then a decrease in the dispersion of the error and an increase in the correlation should be expected. Estimating background noise is a crucial part of the sun radiation observations (as previously stated, we used the term *background noise* to refer to the signal detected at the receiver reference point without any contribution from the sun and a good estimate of background noise is still a difficult task.

**Figure 3:**
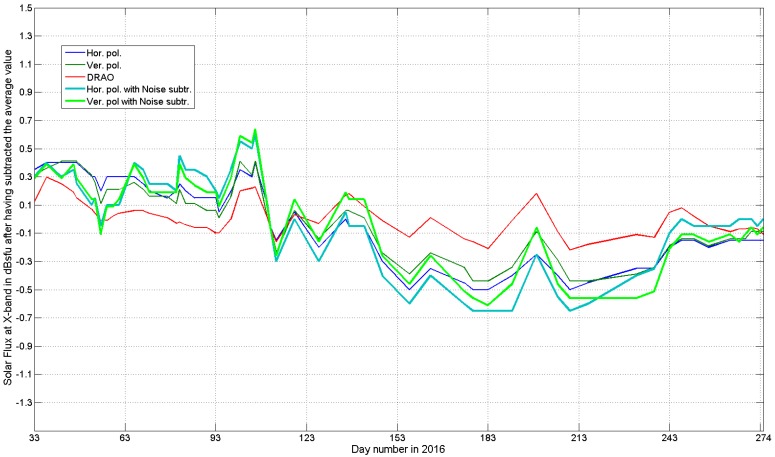
The same curves as in Figure 2, but after having subtracted the average value from each one.

A visual confirmation of our conclusions is presented in Figure 3, which shows solar flux values after having subtracted the average value of each curve. In this way, the DRAO curve shows a zero mean, and the radar curves are all unbiased. It can be seen that, compared to the DRAO reference, the original sun+noise observations (thin lines) are “smoother” and agree better with the DRAO reference, while noise subtraction seems to introduce small “artificial peaks and valleys”. In both cases, the vertical channel estimates agree (slightly) better with DRAO than the horizontal ones. In fact, once the residual bias has been subtracted, it is (even) easier to see that all the radar-retrieved curves capture and reproduce the slowly varying component of the sun, even at 3.2 cm. However, it would be better to compensate atmospheric attenuation before subtracting an average value since the attenuation depends on the angle of elevation of the sun. For instance, attenuation was around 0.22 dB on 2 February, 0.10 on 21 June and 0.16 dB on 30 September.

The issue of well calibrated differential reflectivity is crucial for successful meteorological applications of dual-polarization radar [25]. The sun is a cost/benefit wise tool for calibrating the polarimetric receiving chain: offline sun measurements, in which operational scanning is stopped and the antenna beam axis is pointed towards the center of the sun (hence maximizing the sun-to-noise ratio) have been successfully used at S-band [25] and C-band [9]. National meteorological services prefer to use solar signals “randomly” detected during the operational scan program [12,13,14]: the signal-to-noise ratio is less favorable, but the radar does not need to be offline. In this paper an automatic Sun-tracking scan program that combines the advantages of both approaches is presented. As it can be seen in Table A1, the reception differential bias (independently of DRAO reference values, just considering the ratio between H and V) is +0.06 dB for raw observations and +0.09 dB after noise subtraction. It should be 0.00 dB, because the quiet and slowly varying components of the (thermal) solar radiation have equal power at vertical and horizontal polarizations. Ideally, the variance of H and V should be equal and the (square of the) correlation coefficient between them should be 1. In our real case, the explained variance between H and V (57 samples) is 96.0% for raw data and 92.5% after noise subtraction (see Table 4 for a comparison). It is worth reminding that sun-based checks are useful to characterize only the receiving part of the radar, not the transmitting one.

Similarly, the calibration with respect to the DRAO reference presented in this paper deals solely with the receiving chain of the radar. In order to characterize also the transmitting chain one could use a one-way passive calibrator, for instance the one presented in Section 2.1 in [26]. A calibration of the whole dual-polarization radar system (receiving and transmitting chains) would be the optimal solution, but this task is certainly more complex. The differential reflectivity bias can be (monitored and) assessed using vertical scan and echoes from light rain [27], while absolute calibration of the radar reflectivity is certainly more challenging. Attempts have been made using the idea of self-consistency between reflectivity, differential reflectivity and specific differential phase [25]. Very recently, a multisensor conceptual experiment based on collocated disdrometers, a 24 GHz FMCW Doppler radar and a dual-polarization weather radar under test was able to put in evidence a weather radar underestimation of reflectivity of the order of 1.8 dB [27]. The receivers were checked using the sun and the average bias was found small (~0.2 dB). Consequently, the bias derived using external co-located sensors is attributed to the transmitting chain.

## 6. Conclusions

In conclusion, it is possible to state that the dual-pol receivers of the X-band radar were well calibrated during the 241-day period (2016) in Payerne, in both absolute and relative terms. An absolute calibration cannot, but consider noise-subtraction. As for the relative agreement, the radar was able to reproduce the slowly varying solar component: with 55 degrees of freedom, the correlation coefficient between sun+noise and the DRAO reference is 0.76 (0.67) for the vertical (horizontal) channel. The standard deviation of the difference is 0.18 (0.23) dB for the vertical (horizontal) channel. Our estimate of noise is not optimal; in fact, both the dispersion of the error and the correlation become worse after daily noise subtraction. For this reason, we prefer to use the original data to assess the relative error between H and V regardless of the DRAO reference. In this way, the horizontal channel has on average resulted to be +0.06 (±0.06) dB stronger than the vertical one. It is possible that, in the future, in order to improve the estimation of noise, “Noise scan acquisitions” could be attempted as suggested in the last part of Section 4.2 in [28].

It could also be interesting to investigate the possible causes of the small drifts observed during the 241-day field experiment. Our hypothesis is that the temperature inside the Rx box could play a major role and in our future experiments we plan to measure the temperature inside the antenna-mounted receiver box in and investigate its correlation with the difference between the retrieved signal and the reference, as measured by the DRAO of the Canadian National Research Council.

## Figures and Tables

**Table 1:** Nominal receiver (Rx) Losses and antenna Gain of the X-band weather radar.

Rx Chain Performance	Horizontal Pol.	Vertical Pol.
Rx Losses including radome	2.15 dB	2.25 dB
Antenna Gain	42.6 dB	42.6 dB

**Table 2:** Main statistical parameters of the three curves shown in Figure 2.

	DRAO	N_subtr_ Hor.	N_subtr_ Ver.
Median	24.36 dBsfu	24.00 dBsfu	24.00 dBsfu
Average	24.37dBsfu	23.99 dBsfu	23.91 dBsfu
St. dev.	±0.120 dBsfu	±0.344 dBsfu	±0.321 dBsfu

**Table 3:** Dispersion of the differences between the reference DRAO values and the radar ones.

	Hor.	Ver.	N_subtr_ Hor.	N_subtr_ Ver.
St. Dev. of the difference	0.23 dB	0.18 dB	0.28 dB	0.25 dB

**Table 4:** Explained variance of the retrieved radar values when the DRAO reference measurements are used as the independent variable.

	Hor.	Ver.	Hor. N_subtr_.	Ver. N_subtr_
Explained variance in percentage	44.4%	57.0%	41.1%	55.4%

## Appendix A

This appendix contains both the (DRAO) reference and radar-retrieved solar flux data. The values, which are listed in chronological order in Table A1, are expressed as log-transformed solar flux units (sfu). *S* is the Solar retrieved signal (expressed in solar flux units) and S_0_ represents the normalization factor that is equal to 1 sfu, which is 10^−19^ mW·m^−2^·Hz^−1^. The *s* values, in dBsfu, are then simple to calculate as *s* = 10 Log(*S*/S_0_) is used, where S_0_ = 10^−19^ mW·m^−2^·Hz^−1^. In other words, [*S*] = sfu, while [*s*] = dBsfu.

**Table A1:** DRAO reference and radar retrieved values expressed in dBsfu at the X-band.

Date	Orig. H.	Orig. V.	DRAO	Noise-Sub H.	Noise-Sub V.
2 February (33)	26.10	26.00	24.49 ± 0.015	24.30	24.20
6 February (37)	26.15	26.05	24.67 ± 0.023	24.40	24.30
11 February (42)	26.15	26.10	24.62 ± 0.037	24.30	24.20
15 February (46)	26.15	26.10	24.56 ± 0.009	24.35	24.30
16 February (47)	26.15	26.10	24.52 ± 0.023	24.25	24.20
21 February (52)	26.05	26.00	24.44 ± 0.021	24.10	24.05
22 February (53)	26.05	25.95	24.41 ± 0.011	24.15	24.05
24 February (55)	25.95	25.80	24.36 ± 0.018	23.95	23.80
26 February (57)	26.05	25.90	24.36 ± 0.015	24.10	24.00
28 February (59)	26.05	25.90	24.39 ± 0.016	24.10	24.00
1 March (61)	26.05	25.90	24.41 ± 0.033	24.10	24.05
6 Mach (66)	26.05	25.95	24.43 ± 0.006	24.40	24.30
9 Mach (69)	26.00	25.90	24.43 ± 0.017	24.35	24.20
11 Mach (71)	25.95	25.85	24.41 ± 0.005	24.25	24.10
17 Mach (77)	25.90	25.85	24.38 ± 0.004 ^1^	24.25	24.10
20 Mach (80)	25.95	25.80	24.34 ± 0.009	24.20	24.10
21 Mach (81)	26.00	25.90	24.35 ± 0.007	24.45	24.30
23 Mach (83)	25.95	25.80	24.33 ± 0.004	24.35	24.20
26 Mach (86)	25.90	25.80	24.31 ± 0.005	24.35	24.15
30 Mach (90)	25.90	25.75	24.31 ± 0.023	24.30	24.10
2 April (93)	25.90	25.75	24.27 ± 0.007	24.20	24.10
3 April (94)	25.80	25.70	24.27 ± 0.006	24.15	24.00
7 April (98)	25.95	25.85	24.37 ± 0.030	24.35	24.20
10 April (101)	26.10	26.10	24.57 ± 0.037	24.55	24.50
14 April (105)	26.05	26.00	24.59 ± 0.019	24.50	24.45
15 April (106)	26.15	26.10	24.60 ± 0.011	24.60	24.55
19 April (110)	25.85	25.70	24.38 ± 0.036	24.10	24.00
20 April (111)	25.70	25.65	24.31 ± 0.034	23.95	23.85
22 April (113)	25.60	25.45	24.21 ± 0.003	23.70	23.65
28 April (119)	25.80	25.75	24.40 ± 0.013	24.00	24.05
6 May (127)	25.55	25.55	24.34 ± 0.021	23.70	23.75
15 May (136)	25.75	25.75	24.54 ± 0.066	24.05	24.10
16 May (137)	25.70	25.75	24.51 ± 0.027	23.95	24.05
21 May (142)	25.45	25.45	24.46 ± 0.017	23.95	24.05
27 May (148)	25.70	25.70	24.36 ± 0.014	23.60	23.65
5 June (157)	25.25	25.30	24.24 ± 0.008	23.40	23.45
12 June (164)	25.40	25.45	24.38 ± 0.029	23.60	23.65
23 June (175)	25.30	25.35	24.23 ± 0.006	23.40	23.40
26 June (178)	25.25	25.25	24.21 ± 0.004	23.35	23.35
1 July (183)	25.25	25.25	24.16 ± 0.007	23.35	23.30
9 July (191)	25.35	25.35	24.36 ± 0.030	23.35	23.45
17 July (199)	25.50	25.60	24.55 ± 0.031	23.75	23.85
24 July (206)	25.35	25.40	24.28 ± 0.035	23.45	23.45
28 July (210)	25.25	25.25	24.15 ± 0.007	23.35	23.35
3 August (216)	25.30	25.25	24.19 ± 0.009	23.40	23.35
19 August (232)	25.40	25.30	24.26 ± 0.011	23.60	23.35
25 August (238)	25.40	25.35	24.24 ± 0.010	23.65	23.40
30 August (243)	25.55	25.50	24.42 ± 0.062	23.90	23.70
3 September (247)	25.60	25.55	24.45 ± 0.022	24.00	23.80
7 September (251)	25.60	25.55	24.39 ± 0.004	23.95	23.80
12 September (256)	25.55	25.50	24.32 ± 0.012	23.95	23.75
19 September (263)	25.60	25.55	24.28 ± 0.006	23.95	23.80
22 September (266)	25.60	25.55	24.30 ± 0.014	24.00	23.75
24 September (268)	25.60	25.55	24.30 ± 0.010	24.00	23.80
26 September (270)	25.60	25.60	24.31 ± 0.020	24.00	23.85
28 September (272)	25.60	25.60	24.30 ± 0.008	23.95	23.80
30 September (274)	25.60	25.60	24.26 ± 0.022	24.00	23.85
Average	25.75	25.69	24.37	23.99	23.91

^1^ One unreliable DRAO measurement has been excluded for day 77.
